# Behavioral and neural evidence for masked conceptual priming of recollection

**DOI:** 10.1016/j.cortex.2012.08.008

**Published:** 2013-06

**Authors:** Jason R. Taylor, Luciano G. Buratto, Richard N. Henson

**Affiliations:** MRC Cognition and Brain Sciences Unit, Cambridge, United Kingdom

**Keywords:** Recollection, Familiarity, fMRI, Conceptual priming, Repetition priming

## Abstract

Previous research has found that masked repetition primes, presented immediately prior to the test item in a recognition memory test, increase the likelihood that participants think that the item was present in a previous study phase, even if it was not. This memory illusion is normally associated with a feeling of familiarity, rather than recollection (e.g., as indexed by Remember/Know judgments), and has been explained in terms of an increased fluency of processing the test item, which, in the absence of awareness of the cause of that fluency (i.e., the masked prime), is attributed instead to prior exposure in the study phase. Recently however, we have found that masked conceptual primes (semantically rather than associatively related to the test item) have the opposite effect of increasing Remember but not Know judgments. This result appears difficult to explain in terms of existing theories of recollection and familiarity. Here we report data from a functional magnetic resonance imaging study using the same design, in which we replicate our previous behavioral findings, and find converging evidence for increased activity following conceptual primes in brain regions associated with recollection. This neural evidence supports an account in terms of “true” recollection (for example, conceptual primes reactivating semantically related information that was generated at encoding), rather than an artifact of the mutually-exclusive nature of the Remember/Know procedure.

## 1 Introduction

Over the last three decades since Mandler’s (1980) proposal that recognition memory can be supported by two distinct processes, numerous behavioral dissociations, in healthy individuals, patients with varieties of brain damage, and more recently in other animals, have been interpreted in terms of the processes of “recollection” and “familiarity” (for review, see e.g., Yonelinas, 2002; Aggleton and Brown, 2006). Over the last two decades, dissociations in functional neuroimaging data, using similar paradigms, have also been interpreted in terms of recollection and familiarity (for review, see e.g., Diana et al., 2007; Mayes et al., 2007). However, the strength of these interpretations rests on the validity of the mapping between experimental measures (that give rise to the dissociations) and the theoretical concepts of recollection and familiarity; a mapping that has often been questioned (e.g., Wixted, 2007). Some researchers like Donaldson (1996) and Dunn (2008), for example, have argued that evidence from Remember/Know judgments, Confidence judgments (e.g., ROC curves) and even Source judgments can be re-interpreted in terms of a single dimension of memory strength (i.e., without needing to appeal to qualitatively distinct processes of familiarity and recollection; see recent exchange in *Trends in Cognitive Science*, 2011, Issue *15*). Moreover, the precise nature of the empirical dissociation – for example, a single, double, or cross-over dissociation – has also been questioned, particularly in neuroimaging data where the mapping between hemodynamic measures and theoretical concepts like memory strength, for example, may be nonlinear (Henson, 2006; Squire et al., 2007). Nonetheless, the popularity of the recollection/familiarity distinction is due largely to the convergence of empirical dissociations across a range of paradigms, most of which appear relatively easy to explain in terms of two distinct processes of recollection and familiarity.

In a standard recognition memory paradigm, a series of items are presented in a Study phase (“studied” items), which participants then have to distinguish, when presented again in a later Test phase, from randomly intermixed “unstudied” items that were not presented at Study. As elaborated in other articles in this special issue, recollection in this paradigm generally refers to retrieval (recall) of contextual information that was present at Study, but that is not present at Test. Examples of this contextual information include spatial location of an item, or other thoughts/associations prompted by that item (corresponding to “external” and “internal” “source” information respectively; Johnson et al., 1993). Conversely, familiarity generally refers to a unitary, acontextual signal associated with the test cue itself, owing for example to residual effects of its recent processing in the Study phase (though may also have other causes; see below), which is attributed to the Study phase by the participant.

**Fig. 1:**
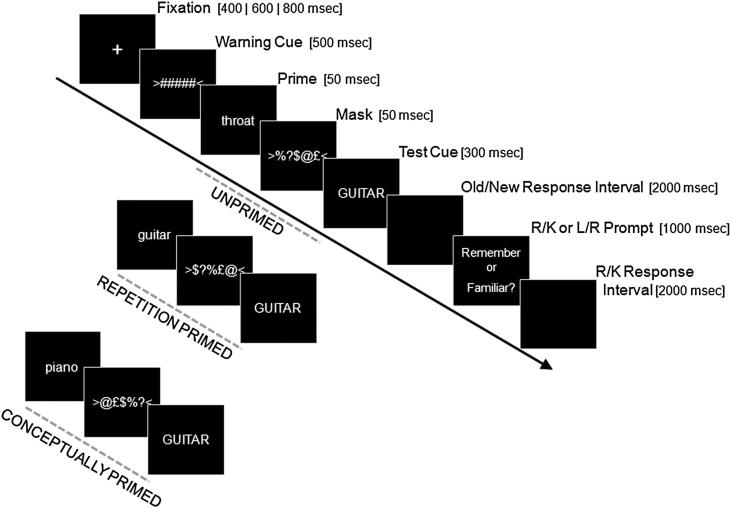
Schematic of trial procedure at Test. Duration of each event is given in square brackets (note that spacing of objects on the timeline does not reflect duration).

One variant of the recognition memory paradigm that has been used to support the recollection/familiarity distinction was introduced by Jacoby and Whitehouse (1989). In the “masked” version of this paradigm, each item in the Test phase is preceded by a brief, masked stimulus, for which participants typically have little to no awareness (or at least, do not appear to spontaneously identify). When the masked stimulus (prime) matches the test item (target), for example corresponding to the same word just in a different letter case (see ahead to Fig. 1), participants are more likely to call the test item “old” (i.e., indicate that it was studied), relative to when the masked stimulus is a different item (unprimed case). Importantly, this increased tendency to call test items “old” typically occurs both for studied items (“hits”) and unstudied items (“false alarms”; FAs). Jacoby and Whitehouse explained this memory illusion in terms of a matching prime increasing the fluency with which a test item is processed, and participants being likely to erroneously attribute this increased fluency, when unaware of its true cause, to the prior Study phase (and hence this could occur for both studied and unstudied items). In support of this hypothesis, when participants were made aware of the prime in a second condition (by increasing the prime duration), this memory illusion actually reversed, such that participants were now less likely to call test items “old” following matching than non-matching primes (which the authors interpreted as participants now sometimes erroneously attributing the fluency induced by study to the prime instead; though see Klinger, 2001; Higham and Vokey, 2004, for alternative explanations for the precise role of awareness/attention).

Though Jacoby and Whitehouse's original findings did not specifically address the familiarity/recollection distinction, a later variant by Rajaram (1993; see also Kinoshita, 1997; Woollams et al., 2008) asked participants for a Remember (R)/Know (K) judgment after each “old” decision to words (Tulving, 1985). Rajaram found that the increase in “old” judgments following masked, matching prime words was restricted to K judgments (i.e., the prime manipulation had no detectable effect on trials given R judgments). This finding is relatively easy to explain according to the recollection-familiarity distinction: The fluency with which test words are processed can be used as an acontextual familiarity signal; whereas one would not expect this fluency to affect people's abilities to recall unrelated contextual information from the Study phase.

The majority of studies combining masked primes with R/K judgments, such as Rajaram’s (1993) original study, have used repetition primes. Priming effects on familiarity in these studies are typically attributed to increased *perceptual* fluency, despite the fact that repetition primes and targets, though the same word, are often presented in different case or font; i.e., are perceptually only similar, and only identical at higher levels of representation (e.g., orthographic, phonological, conceptual, etc.). A later study of Rajaram and Geraci (2000) used semantic primes (e.g., sugar-SWEET, author-BOOK) and found the same priming-related increase in K but not R judgments, suggesting that the familiarity signal arises at the level of conceptual (rather than perceptual, orthographic, or phonological) fluency. However, because Rajaram and Geraci's prime-target pairs were also *associatively* related—i.e., targets were high-probability free-associates of primes—it is possible that the increase in familiarity was due to lexical rather than conceptual fluency. Although many associated words are also conceptually related, as indeed Rajaram and Geraci's were, associative probability is influenced by non-conceptual factors such as the probability of co-occurrence in language (e.g., hobby-HORSE, grand-PIANO), and in semantic priming studies, association tends to dominate over conceptual relatedness (Lucas, 2000).

In a recent study (Taylor and Henson, in press), we used semantically related primes (that share semantic attributes, e.g., piano-GUITAR) that were not associatively related, in an attempt to isolate the effect of conceptual fluency on recognition memory judgments. When we included these so-called conceptual primes with the standard repetition primes used in most previous studies (with different blocks for each prime-type), we found that they produced the opposite effect: i.e., Conceptual primes increased the likelihood of (correct) R but not K judgments.^1^ This occurred simultaneously with the standard increase in K but not R judgments following repetition primes, producing a reliable cross-over interaction between prime-type and R/K judgment.

While this cross-over interaction might be used to support at least two distinct contributions to recognition memory, such as recollection and familiarity, the interpretation of the increased R judgments following conceptual primes would appear more difficult to reconcile with conventional theories of recollection. Indeed, as noted above, one popular theory of recollection and familiarity associates conceptual fluency with familiarity, not recollection (Yonelinas, 2002).

One possibility is that conceptual primes automatically activate concepts that are semantically related to both the prime and target (test item), consistent with behavioral evidence for subliminal semantic priming (Van den Bussche et al., 2009). If some of these concepts were also generated spontaneously at Study (particularly if the encoding task entails semantic elaboration), then their unconscious activation at Test may increase the probability of retrieving them in response to the test cue (i.e., increase retrieval of internal source; the type of source that is likely to dominate R judgments in experiments like these that use word lists, where there is little variability in external source information). In support of this hypothesis, the increase in R judgments following conceptual primes occurred only for studied items (Hits), not unstudied items (False Alarms), unlike the typical pattern for repetition primes (that increase both Hits and False Alarms, given a K judgment) – see Taylor and Henson (in press) for further discussion.

However, another possibility is that this interaction pattern is an artifact of the standard R/K procedure, in that participants are forced to give either an R judgment or a K judgment (i.e., the response categories are mutually exclusive). Thus if participants experience two different types of fluency within an experiment (stemming from repetition versus conceptual primes), then they may feel obliged to attribute one to “knowing” and one to “remembering”. While this account does not explain why conceptual primes lead specifically to R judgments (and only for studied items), it might explain why we have not yet found reliable evidence of increased R judgments in experiments that use conceptual primes only (i.e., with no repetition primes in other blocks; Taylor and Henson, in press). More importantly, this account is consistent with other experiments that have used the Jacoby and Whitehouse paradigm, but asked for independent ratings of both Remembering and Knowing on each trial (e.g., using a 1–4 scale for each; an alternative procedure introduced by Higham and Vokey, 2004). These experiments, by Kurilla and Westerman (2008), and Brown and Bodner (2011), replicated the finding that masked repetition primes only affect K judgments under the standard (exclusive) R/K procedure, but found that they affected both R and K ratings under the independent ratings procedure. In other words, even masked repetition primes (not just conceptual primes) appear to increase participants' experiences of Remembering, as long as participants are allowed to rate this independently of their experience of Knowing.

If one hypothesizes that the processes of recollection and familiarity are mutually exclusive (e.g., Gardiner et al., 1998, 2002), then the use of binary R/K response categories follows naturally; however, if one believes that recollection and familiarity are independent or redundant (e.g., Knowlton and Squire, 1995; Mayes et al., 2007), then the interpretation of binary R/K responses becomes less straightforward. In the latter case, measures such as “independence” K scores (the proportion of trials *not* given an R response that were given a K response; Yonelinas and Jacoby, 1995) may be computed in order to estimate recollection and familiarity from binary R/K responses. Nonetheless, the critical concern here is the signal sent to the participant by the use of binary response categories – that Remembering and Knowing are mutually-exclusive experiences – the effects of which cannot be removed statistically.

One alternative way to test these mappings is to look for convergent evidence from neuroimaging. A large number of functional magnetic resonance imaging (fMRI) experiments have investigated the brain regions associated with many different operationalizations of recollection and familiarity: Not just using R/K judgments, but also using objective tests of source retrieval, confidence ratings, and other means. A notably consistent set of regions has emerged in relation to recollection, *viz* regions in medial and lateral parietal cortex (Wagner et al., 2005) and in the hippocampus (Diana et al., 2007). In the present paradigm, if conceptual primes truly increase recollection of internal source, as in the first possibility outlined above, then they should affect the fMRI signal in these “recollection” regions.

We therefore conducted a replication of our prior behavioral experiment using conceptual and repetition primes in an R/K paradigm (Taylor and Henson, in press) in combination with fMRI. For the fMRI data, differences between the various trial-types (as a function of R/K/New judgments and prime-type) were explored in a whole-brain analysis. Second, as a more sensitive test of the hypothesis above, we identified functional regions of interest (fROIs) sensitive to recollection (R > K contrast) or to familiarity (K > Correct Rejections (CR) contrast), and tested for (orthogonal) priming effects in those fROIs.

## 2 Materials and methods

### 2.1 Participants

Participants were recruited from the volunteer panel of the MRC Cognition and Brain Sciences Unit, or from the student population of Cambridge University; all participants had normal or corrected to normal vision and were right-handed (self-report). Experiments were of the type approved by a local research ethics committee (Local Research Ethics Committee reference 05/Q0108/401). A total of 22 participants (15 female) gave informed consent to participate in the fMRI experiment, with a mean age of 25.77 (SD = 4.57) years.

### 2.2 Materials

The stimuli (identical to those used in Taylor and Henson, in press) consisted of 480 word-pairs (“prime”-“TARGET”) that were conceptually related but not lexically associated according to word-generation norms (both forward and backward association probabilities <.10 in the University of South Florida norms; Nelson et al., 1998: http://www.usf.edu/FreeAssociation/). Conceptual relatedness was defined on the basis of taxonomic category (e.g., piano–GUITAR, horse–COW), attributes or functions (e.g., silver–COIN, teapot–BOIL), typical context (e.g., pond–FROG, wedding–BRIDE), part-whole relationship (e.g., tobacco–CIGAR, camera–LENS), or lexical interchangeability (e.g., biscuit–COOKIE, shop–BOUTIQUE). All primes and targets were between three and eight letters long (primes: *M* = 5.26, *SD* = 1.12; targets *M* = 5.44, *SD* = 1.38) and had written frequencies between 1 and 150 per million (primes: *M* = 33.97, *SD* = 26.00; targets *M* = 34.14, *SD* = 36.08; Kučera and Francis, 1967). These conceptually related prime-target pairs comprised the Primed condition for Conceptual Priming trials; two further lists were created by re-pairing each target with itself (Primed condition, Repetition Priming trials) or with an unrelated prime via a pseudo-random shuffle (Unprimed conditions for both Conceptual and Repetition Priming trials). These lists were each further sub-divided into four Sets (A–D), to be used in the counterbalancing described in Procedure, below.

### 2.3 Procedure

The main experiment consisted of two trial types, Study and Test. On Study trials, participants made “interestingness” judgments (based on our previous studies, e.g., Woollams et al., 2008): A word (in upper case) was presented at the center of the screen (duration 300 msec) and participants read the word silently and indicated whether it was *interesting* or *not* by pressing one of two buttons. The word was preceded by a central fixation cross for one of 400, 600, or 800 msec (selected from a random uniform distribution) and followed by a blank screen for 1000 msec.

On Test trials, participants performed a yes/no recognition task: They read a centrally presented word (see Fig. 1 for stimulus timing) and first indicated whether they thought it had (*old*) or had not (*new*) appeared previously in a Study trial. If they responded “old”, they were then prompted to decide whether they *remembered* seeing the test cue (“R” judgment) or whether they simply felt that the item was *familiar* (“K” judgment; instructions are described below). Note that we used the label “familiar”, rather than the traditional “know” judgment, for reasons given in Footnote 1. Response times (RTs) were recorded; however, accuracy was emphasized over speed. If the participant responded “new” to the test cue, or if they failed to respond (time limit = 2000 msec), then they were prompted (“Left/Right”) to randomly press one of the response keys. This helped match the timing and motor demands of “old” and “new” trials (over which the fMRI response averages). Critically, each test cue (“target”) was preceded by a brief, masked prime word. In the Conceptual Priming condition, the prime and target were either conceptually related or unrelated; in the Repetition Priming condition, the prime and target were either the same word or unrelated words, as described in Stimuli, above. Primes were presented in lower case and targets in upper case, to minimize visual overlap on Repetition priming trials.

Before entering the MRI scanner, participants were given task instructions and completed a brief practice session (eight Study and 16 Test trials). The instructions, based on Rajaram (1993), described the Remember/Familiar distinction as follows: “Respond REMEMBER if you recollect the event of seeing the word, some aspect of the context (how the word looked, what it made you think or feel, etc.). Respond FAMILIAR if you are certain you saw the word previously but you cannot recollect any contextual details.” At the end of the practice trials, participants were asked to recall the instructions and explain the difference between the Remember and Familiar response categories; any confusion was resolved by repeating the relevant part of the instructions. For example, if the participant seemed to equate Remember/Familiar responses with high/low confidence, the experimenter suggested that high-confidence Familiar responses were possible, such as when one is sure the word was presented previously but no contextual details of the event of seeing the word could be recalled.

The experiment consisted of four cycles of interleaved Study and Test blocks, all conducted during functional MRI scanning. Each Study block (duration: approximately 2.5 min) consisted of 60 trials (order randomized for each participant) plus two “dummy” trials at the end (ignored in analysis) to prevent recency effects. Each Test phase (duration: approximately 11 min) consisted of 120 trials (50% = 60 trials/block “studied” words from the previous Study phase, 50% “unstudied” words that had not been presented in the experiment; order randomized for each participant) plus two “practice” trials at the beginning (unstudied words; ignored in analysis). One half of studied trials and one half of unstudied trials were preceded by related primes; the other halves were preceded by unrelated primes. The Conceptual and Repetition priming conditions were blocked such that two consecutive Test phases contained either Conceptual primes or Repetition primes. No word was repeated across blocks.

Block Order (Repetition/Conceptual Priming first) and Set-Condition mapping (A/B/C/D → Repetition/Conceptual × Primed/Unprimed) were counterbalanced across participants, with a total cycle of eight participants. Stimuli were back-projected (60 Hz refresh rate; 1024 × 768 pixels) onto a screen behind the MRI scanner that participants viewed through a mirror. Words were presented in white on a black background. Responses were made with right and left index fingers, with finger-response mappings separately counterbalanced across participants for the Interestingness, Old/New, and R/K tasks.

On completion of the main experiment, subjective and objective measures of prime awareness/visibility were collected. Participants were asked whether they noticed any “hidden words” (i.e., the masked primes) in the procedure, and whether they had been able to identify any of these words (subjective measures). The nature of the experiment, and in particular of the masked primes, was then explained. Participants then performed a Prime Visibility Test, in which 120 test trials were shown as during the experiment (fixation, forward mask, prime, backward mask, test cue), and participants were asked to indicate which of three (equally likely to be correct across trials) candidate words had been the prime on that trial. The three candidate primes were (a) the same word as the target (i.e., the Repetition prime), (b) a conceptually related word (i.e., the Conceptual prime), and (c) an unrelated word (Unprimed condition). Participants were encouraged to guess if they didn't see the prime.

### 2.4 Behavioral analyses

Recollection and familiarity were estimated from proportions of trials given “remember” and “familiar” judgments under independence assumptions (“IRK”; Yonelinas and Jacoby, 1995), where recollection = R/N and familiarity = K/(N–R); R = number of R judgments; K = number of K judgments and N = total number of test trials. Separate estimates were made for studied (i.e., hits) and unstudied (i.e., Correct Rejection) trials, and for each priming condition. These estimates were analyzed using a multifactorial repeated-measures analysis of variance (ANOVA). The purpose of using this IRK scoring was to allow Memory Judgment (R, K) to be used as a factor in the same ANOVA, given that interactions between Priming and Memory Judgment were of primary interest. Significant effects are only reported in the absence of significant higher-order interactions. All statistical tests had alpha set at .05, and a Greenhouse–Geisser correction was applied to all F-values with more than one degree of freedom in the numerator. Follow-on *T*-tests were two-tailed, except where stated otherwise. An ANOVA on RTs to the first (old/new) decision was also conducted, though note that participants' responses were not speeded, so any RT effects (and in particular their absence) should be interpreted with caution.

### 2.5 fMRI methods

#### 2.5.1 fMRI acquisition

Thirty-two T2*-weighted transverse slices (64 × 64 3 mm × 3 mm pixels, TE = 30 msec, flip-angle = 78°) per volume were taken using Echo-Planar Imaging (EPI) on a 3T TIM Trio system (Siemens, Erlangen, Germany). Slices were 3-mm thick with a .75 mm gap, tilted up approximately 30° at the front to minimize eye-ghosting, and acquired in descending order. Eight sessions were acquired, equating to the four study-test cycles. Seventy-six volumes were acquired during each Study phase, 340 were acquired during each Test phase, with a repetition time (TR) of 2000 msec. The first five volumes of each session were discarded to allow for equilibrium effects. A T1-weighted structural volume was also acquired for each participant with 1 × 1 × 1 mm voxels using Magnetisation Prepared Rapid Gradient Echo (MPRAGE) and Generalized Autocalibrating Partially Parallel Acquisition (GRAPPA) and GRAPPA parallel imaging (flip-angle = 9°; TE = 2.00 sec; acceleration factor = 2). fMRI data were acquired during all phases of the experiment; analyses presented here are limited to Test Phase data.

#### 2.5.2 fMRI analyses

fMRI data were analyzed using Statistical Parametric Mapping (SPM5, http://www.fil.ion.ucl.ac.uk/spm5.html). The EPI volumes were realigned spatially to correct for movement, and then the data within each slice were realigned temporally to match acquisition of the middle slice. The mean EPI across realigned volumes was then coregistered to the T1 image, which was normalized to MNI space, using a unified segmentation and normalization algorithm (Ashburner and Friston, 2005); the resulting normalization parameters were then applied to all of the EPI images, which were resampled to 3 × 3 × 3 mm voxels. Finally, the normalized EPI images were smoothed with an isotropic Gaussian kernel with 8 mm full width at half maximum (FWHM; final smoothness approximately 10 × 10 × 10 mm).

Statistical analysis was performed in a two-stage approximation to a Mixed Effects model. In the first stage, neural activity was modeled by a delta function at stimulus onset. The BOLD response was modeled by a convolution of these delta functions by a canonical Hemodynamic Response Function (HRF). The resulting time-courses were down-sampled at the midpoint of each scan to form regressors in a General Linear Model.

For each Test session, 12 regressors were modeled, corresponding to six types of response (R Hits, R Misses, K Hits, K Misses, Correct Rejections, False Alarms) for each of Primed and Unprimed trials (the manipulation of Repetition versus Conceptual Prime-Types was across sessions). To account for (linear) residual artifacts after realignment, the model also included six further regressors representing the movement parameters estimated during realignment. Voxel-wise parameter estimates for these regressors were obtained by Restricted Maximum-Likelihood (ReML) estimation, using a temporal high-pass filter (cut-off 128 sec) to remove low-frequency drifts, and modeling temporal autocorrelation across scans with an AR (1) process (Friston et al., 2002).

##### 2.5.2.1 Whole-brain analyses

Voxel-wise contrasts of the parameter estimates for each of the 12 event-types of interest, conforming to the 3 × 2 × 2 design of Memory Judgment (R Hits, K Hits, Correct Rejections) × Priming Type (Repetition, Conceptual) × Prime Status (Primed, Unprimed), were estimated by a weighted average (*vs*baseline) across each of the two sessions per Prime Type, weighted by the number of events of that type across those two sessions. The resulting contrast images comprised the data for a second-stage model, which treated participants as a random effect. Within this model, Statistical Parametric Maps (SPMs) were created of the *T*-statistic for the various effects of interest, using a single pooled error estimate for all contrasts, whose nonsphericity was estimated using ReML as described in Friston et al. (2002). The SPMs were thresholded for at least five contiguous voxels whose statistic exceeded a peak threshold corresponding to one-tailed *p* < .05 family-wise error-corrected across the whole space using Random Field Theory (RFT). Stereotactic coordinates of the maxima within the thresholded SPMs correspond to the MNI template.

##### 2.5.2.2 fROI analyses

To provide a more sensitive test of possible priming effects, the same 3 × 2 × 2 ANOVA was conducted on data from the peak voxel within each fROI defined in whole-brain comparisons of Memory Judgment. As the main effect of Memory Judgment is biased by the selection of voxels, only effects involving Prime Status or Priming Type factors are reported.

## 3 Results

### 3.1 Behavioral results

#### 3.1.1 Priming and memory judgments

The mean proportions of responses in each condition are shown in Table 1. For R judgments, overall accuracy (Pr[Hit-FA]) was .56 in Conceptual Priming and .58 in Repetition Priming blocks, both significantly greater than zero, *t*(21)s > 10.0, *p*s < .001. For independent scoring of K judgments (see Methods), accuracy was .29 in Conceptual Priming and .31 in Repetition Priming blocks, both of which were also significantly above chance, *t*(21)s > 5.5, *p* < .001, suggesting that K judgments were not simply guesses.

**Fig. 2:**
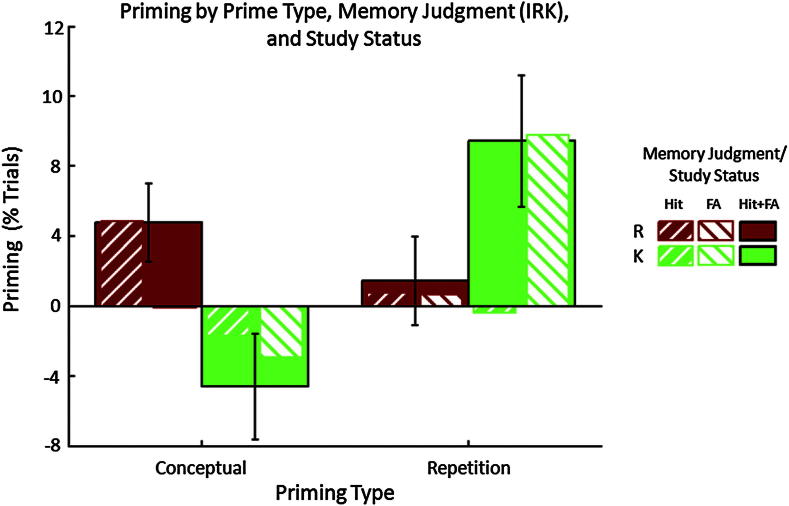
Behavioral priming effects. Priming score (percentage of trials for Primed-Unprimed, under independence assumptions) is shown as a function of Memory Judgment (R – red, K – green) and Priming Type (Conceptual, Repetition), while superimposed hatched bars show further split by Hits versus False Alarms. Error bars reflect standard error of the mean priming effect.

For “old” judgments, the 2 (Memory Judgment) × 2 (Priming Type) × 2 (Study Status) × 2 (Prime Status) ANOVA revealed several significant 3-way interactions, each involving the Prime Status factor (i.e., priming effects). Most importantly, the Priming Type × Memory Judgment × Prime Status interaction, *F*(1,21) = 5.05, *p* = .035, indicated that the pattern of R- and K-priming effects differed between Conceptual and Repetition Priming blocks. Follow-up *t*-tests on priming score (Primed-Unprimed) showed that masked Conceptual primes increased R judgments [*t*(21) = 2.13, *p* < .05] but not K judgments [*t*(21) = −1.53, *p* = .14], whereas masked Repetition primes increased K judgments [*t*(21) = 2.52, *p* = .02] but not R judgments [*t*(21) = .57, *p* = .57].^2^ This cross-over interaction, as shown in Fig. 2, replicates our previous behavioral experiment (Taylor and Henson, in press).

Further three-way interactions were found for Priming Type × Study Status × Prime Status, *F*(1,21) = 18.9, *p* < .001, and for Memory Judgment × Study Status × Prime Status *F*(1,21) = 8.52, *p* = .008. These effects together indicated that the pattern of R- and K-priming effects differed between Studied (Hits) and Unstudied (FAs) items. Follow-up *t*-tests on priming score revealed that Conceptual primes increased R Hits [*t*(21) = 2.47, *p* < .05] but not R FAs (*t* < 1), whereas Repetition priming increased K FAs [*t*(21) = 4.31, *p* < .001] but not K Hits (*t* < 1).^3^

#### 3.1.2 RT

Median RTs for correct “old” (Hit) and “new” (CR) decisions (there were too few False Alarms and Misses to include these) were analyzed in a 2 × 3 × 2 ANOVA with factors Priming Type (Conceptual, Repetition), Memory Judgment (R, K, CR), and Prime Status (Primed, Unprimed). Participants were excluded from the analysis if they had an insufficient number of trials in each cell of the design, using the same criteria as in the fMRI analysis (see section 3.2.1 below), i.e., the same sample of 18 participants used in fMRI Results.

The ANOVA revealed a main effect of Memory Judgment, *F*(1.89,32.19) = 11.1, *p* < .001, and follow-up *t*-tests showed that RTs to correct “old” decisions subsequently given an R judgment (*M* = 752 msec, *SD* = 98) were significantly faster than those subsequently given a K judgment (*M* = 865 msec, *SD* = 195), *t*(17) = 4.27, *p* < .01. Such Rs were also significantly faster than CRs (*M* = 808 msec, *SD* = 151), *t*(17) = 2.38, *p* < .05, and CRs were faster than Ks, *t*(17) = 2.64, *p* < .05. The main effect of Prime Status was not significant (*F* < 1); however, the interaction between Memory Judgment and Prime Status was significant, *F*(1.98,33.6) = 4.26, *p* < .05, and follow-up *t*-tests showed that the priming effect (Primed-Unprimed, collapsed across Conceptual and Repetition blocks) was significantly larger for R (*M* = 35 msec) than for CR (*M* = −9 msec), *t*(17) = 2.98, *p* < .01, and nearly significantly larger than the priming effect for K (*M* = 3 msec), *t*(17) = 1.97, *p* = .065. Only the priming effect on Rs was significantly greater than zero, *t*(17) = 4.65, *p* < .001.

#### 3.1.3 Prime Visibility Test

Nine of the 22 participants (41%) reported being aware that there were “hidden” words in the experiment; only one of these “aware” participants reported being able to identify prime words on some trials. In the Prime Visibility Test, mean performance was 58.7% (*SD* = 16.5), which was significantly better than chance (33%), *t*(21) = 7.30, *p* < .001. However, performance varied greatly between priming conditions (one-way ANOVA with factor Prime Type: Conceptual, Repetition, Unrelated; *F*(1.47,30.8) = 13.0, *p* < .001). Participants identified both Unrelated (*M* = 67.6%, *SD* = 27.1) and Conceptual (*M* = 74.5%, *SD* = 19.8) primes with greater accuracy than Repetition primes (*M* = 34.0%, *SD* = 35.8), *t*(21)s > 3.4, *p*s < .01. Indeed, prime identification accuracy did not significantly differ from chance for Repetition primes, *t*(21) = 1.30, *p* = .21, but was greater than chance for both Conceptual and Unrelated primes, *t*(21)*s* > 5, *p*s < .001.

### 3.2 fMRI results

#### 3.2.1 Whole-brain analyses

The fMRI data of four participants were excluded (leaving 18) because they did not produce at least one event of each of the 12 event-types of interest (conforming to the 3 × 2 × 2 design of Memory Judgment: R Hits/K Hits/Correct Rejections × Priming Type: Repetition/Conceptual × Prime Status: Primed/Unprimed, as also used for RTs above), precluding estimation of BOLD responses in those conditions (see ranges in Table 1). We started with directional, pairwise T-contrasts of different Memory Judgments, in order to replicate previous fMRI studies using R/K judgments (e.g., Henson et al., 1999; Eldridge et al., 2000). The results are shown in Table 2.

**Fig. 3:**
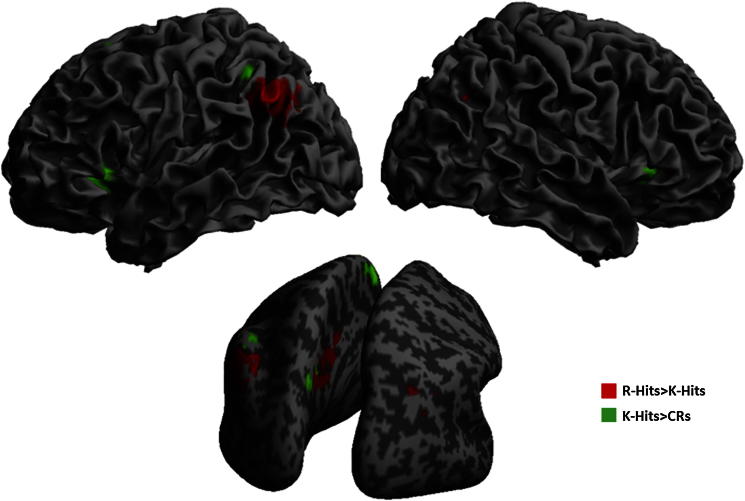
Rendering of regions showing greater BOLD signal for R Hits than K Hits (red) and for K Hits than CRs (green) at *p* < .05 corrected for a whole-brain search, shown on left and right views (upper row) and posterior view (lower image) of the surface of a canonical brain in MNI space (slightly inflated in the lower image in order to help visualization of activity in medial sulci).

The regions showing significantly greater activity for R Hits than K Hits are shown in red in Fig. 3, whereas regions showing greater activity for K Hits than CRs are shown in green. As expected from previous studies, R-related activity occurred in medial and lateral parietal cortex, particularly bilateral posterior cingulate and inferior parietal gyri respectively (no voxels survived correction in the hippocampi; though see fROI results below). Greater activity for K Hits than Correct Rejections, on the other hand, included more posterior regions of medial parietal cortex and more superior regions of lateral parietal cortex, consistent with the review of Wagner et al. (2005), as well as bilateral anterior cingulate and anterior insulae. These K > CR regions were generally activated by Hits, regardless of R or K judgment (see fROI results below, Fig. 5C).

**Fig. 4:**
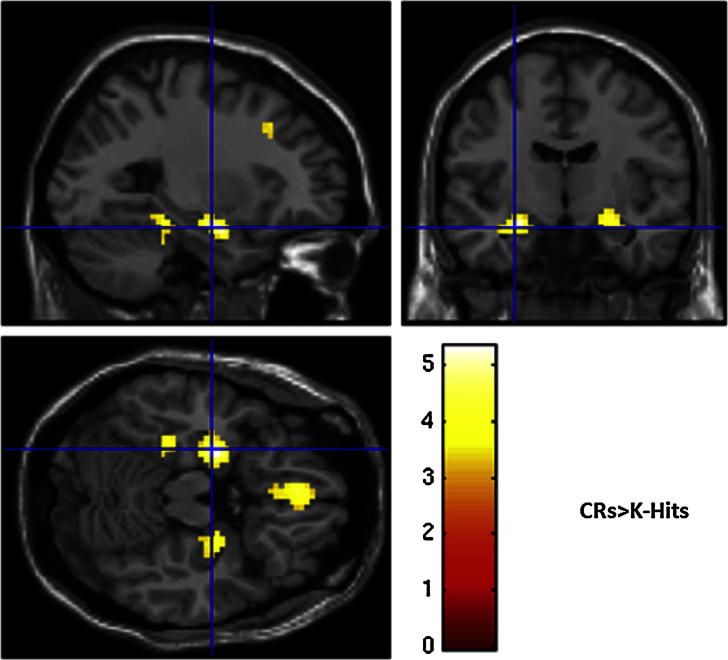
Sections through a canonical T1-weighted image in MNI space showing hippocampal regions that demonstrated greater BOLD signal for CRs than K Hits at *p* < .001 uncorrected for display purposes (maximum in left hippocampus survives *p* < .05 corrected for the whole-brain).

For the reverse contrasts, no region showed significantly greater activity for K Hits than R Hits. However one region, in left anterior hippocampus, showed significantly greater activity for Correct Rejections than K Hits (at a lower statistical threshold, a homologous region in the right hippocampus was also revealed; see Fig. 4). This is consistent with the “novelty” response often seen in hippocampus with fMRI (Daselaar et al., 2006; Köhler et al., 2005; Yassa and Stark, 2008), though its full response pattern was more complex (see fROI analysis below).

We also tested using F-contrasts the various main effects and interactions involving Prime Status and Priming Type in the 2 × 2 × 3 ANOVA design. However, no voxels survived corrections for multiple comparisons across the whole-brain. Therefore, we next focused on fROI defined by the above contrasts of R Hits, K Hits and Correct Rejections.

#### 3.2.2 fROI results

To provide a more sensitive test of possible priming effects, we repeated the 2 × 2 × 3 ANOVA on data from the peak voxel within each fROI defined in the whole-brain comparisons of Memory Judgment above. The main effect of Memory Judgment is, of course, biased by the selection of voxels, so we only report on effects involving Prime Status or Priming Type factors.

For the three fROIs that were more active for R Hits > K Hits (Table 2), two (in left and right inferior parietal cortex) showed a significant interaction between Priming Type and Prime Status [*F*(1,17)*s* > 5.3, *p*s < .05], while the third (in posterior cingulate cortex) showed a trend in the same direction [*F*(1,17) = 3.27, *p* = .09]. No other effects of interest reached significance. When including fROI as an additional factor, the Priming Type and Prime Status was again significant [*F*(1,17) = 6.90, *p* < .05], as was a main effect of Priming Type [*F*(1,17) = 7.01, *p* < .05], but no other effects reached significance, including any interactions with fROI.

**Fig. 5:**
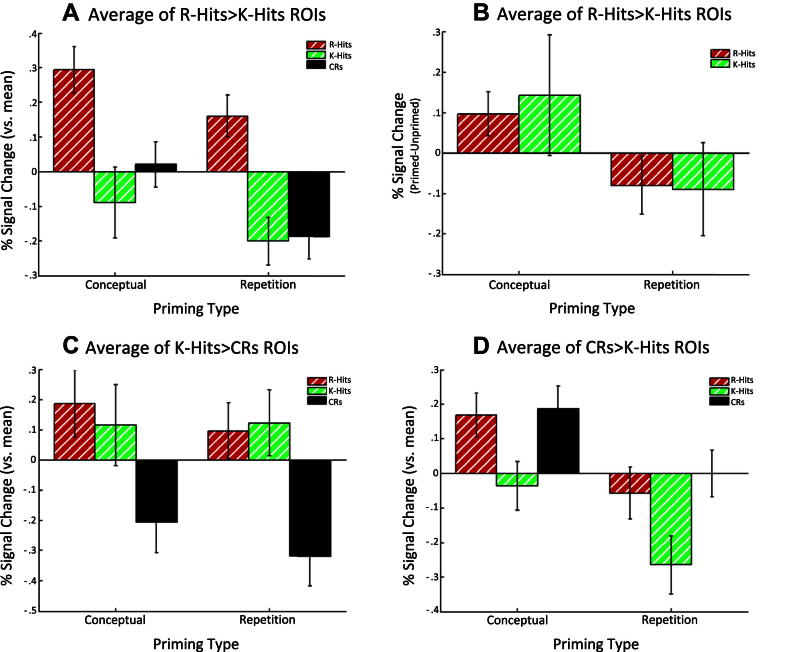
Plots of mean BOLD signal change for significant ANOVA effects averaged across ROIs in Figs. 2, 3. Note: effect of Memory Judgment in panels A, C and D is illustrative only, since voxel maxima were selected after searching many voxels for a reliable difference between two of the three conditions Memory Judgments (Kriegeskorte et al., 2010), but this does not bias the orthogonal effect of priming shown in panel B.

The associated BOLD signal changes, averaged across these parietal “remember fROIs” are shown in Fig. 5A–B. Fig. 5A shows the effects of Memory Judgment for each Priming Type (averaged across Prime Status), though note that these plots are for illustrative rather than inferential purposes, given the prior selection of these fROIs as showing (part of) an effect of Memory Judgment (Kriegeskorte et al., 2010). From this figure, it can be seen that while these regions distinguish R Hits from the other judgment types, there is little evidence for a difference between K Hits and Correct Rejections (i.e., these parietal regions seemed interested specifically in R judgments).

**Fig. 6:**
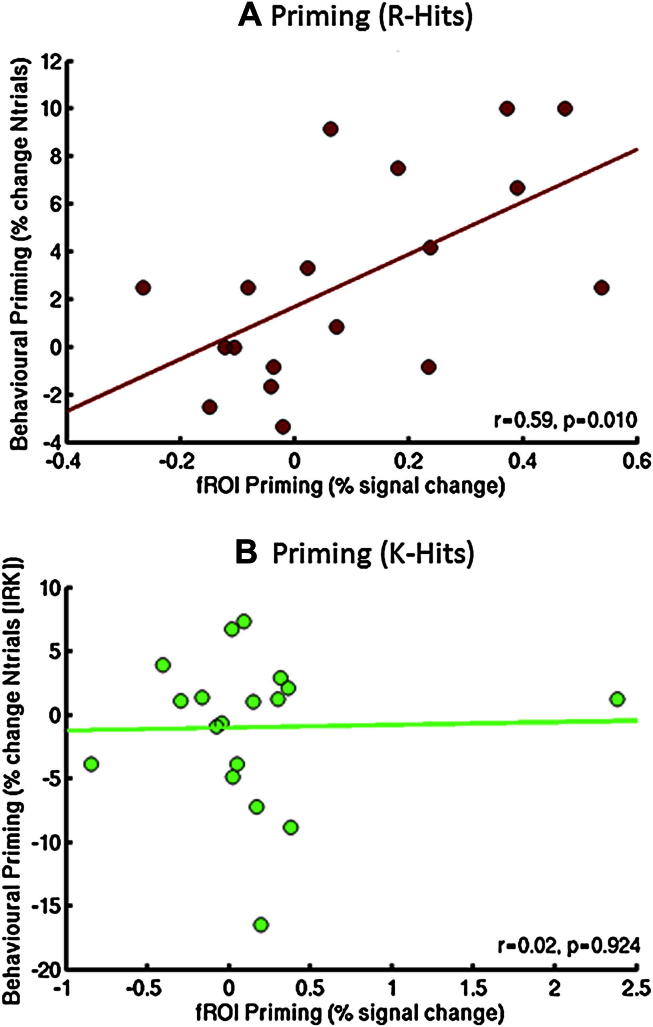
Plots of behavioral priming scores (Primed-Unprimed) for correct “old” judgments (Hits; under independence assumptions) against BOLD priming effects (Primed-Unprimed) averaged across R Hit > K Hit fROIs for R (Panel A) and K (Panel B) judgments. Note the outlier in Panel B (see Footnote 4).

Fig. 5B, on the other hand, shows the effects of Priming Type on the priming effect, separately for R and K Hits (analogous to the format of behavioral priming effects used in Fig. 2, but only for trials correctly identified as “old”, i.e., Hits). This figure, which is not biased by selection by the orthogonal main effect of Memory Judgment, demonstrates opposite effects of Repetition and Conceptual priming on the BOLD signal in the “Remember ROIs”, corresponding to the significant interaction between Priming Type and Prime Status in the above fROI ANOVAs. Unlike the behavioral data, however, this effect of Priming Type appears relatively unaffected by Memory Judgment (i.e., does not differ for R and K),^4^ though it is worth noting that only the increased response for Primed relative to Unprimed Conceptual trials is independently significant [*t*(17) = 1.78, *p* < .05], which may relate to the behavioral increase for Conceptual priming that was specific to R judgments (Fig. 2). Indeed, even more strikingly, the Conceptual priming effect for R in these regions correlated significantly with behavioral priming of R judgments, *r* = .59, *p* = .01, whereas no such correlation was found between behavioral and BOLD K-priming effects in these “Remember” regions (consistent with expectation), *r* = .02, *p* = .92 (Fig. 6).

For the five fROIs that were more active for K Hits > Correct Rejections (Table 2), only one showed a significant effect involving Priming Type or Prime Status, and this was the fROI in right anterior insula, which showed a significant main effect of Prime Status [*F*(1,17) > 5.1, *p* < .05], though this may be a Type I error given the number of ANOVA effects and fROIs tested. More importantly, when averaging across these five “familiarity fROIs”, no effects involving Prime Status or Priming Type reached significance (*F*s < 2.47, *p*s > .14). Thus these regions seemed to care only about the Memory Judgment, as shown for illustrative purposes in Fig. 5C, from which it appears that these regions distinguish Hits from Correct Rejections, regardless of whether Hits are associated with R of K judgments.

Finally, for the single left hippocampal fROI that was more active for Correct Rejections than K Hits, the ANOVA showed no significant effects involving Prime Status or Priming Type except a main effect of Priming Type [*F*(1,17) = 7.90, *p* < .05], which reflected greater overall activity in Conceptual Priming than in Repetition Priming blocks (Fig. 5D).^5^ Interestingly, and in keeping with many previous fMRI studies using the R/K procedure in our laboratory, this anterior hippocampal region showed a pattern across Memory Judgments that appeared to differ from both of the above two types of fROI: a “U-shaped” pattern such that the hippocampus was most active for Correct Rejections and R Hits relative to K Hits. An explanation for this pattern is given in the Discussion.

## 4 Discussion

In a previous behavioral study (Taylor and Henson, in press), we found that masked conceptual primes increase the number of R but not K judgments, whereas masked repetition primes produce the opposite pattern, increasing K but not R judgments. If the effect of conceptual priming on R reflects a genuine influence of conceptual primes on recollection, rather than an artifact of the binary response demands of the R/K procedure (Brown and Bodner, 2011; Kurilla and Westerman, 2008), then conceptual priming would be expected to modulate activity in neural regions that support recollection. In the present fMRI study, we replicated the behavioral finding that conceptual priming increases R judgments, and further, we found that conceptual priming did indeed modulate BOLD responses in medial and lateral parietal regions that were sensitive to recollection (identified via a whole-brain contrast of R Hits > K Hits), and that the magnitude of parietal fROI priming effects correlated with behavioral priming effects across participants. In what follows, we expand some details and alternative interpretations of the behavioral and fMRI results, integrate the fMRI results with those of previous studies of recognition memory, and finally, present some potential caveats concerning the present analyses.

### 4.1 Masked conceptual primes and recollection

While the cross-over interaction between conceptual versus repetition primes and R versus K judgments on behavioral priming (the difference in number of R/K judgments for primed versus unprimed trials) replicates our previous results (Taylor and Henson, in press), there were a few differences in the precise pattern. Firstly, the effect of repetition primes on K judgments was significantly greater for False Alarms than Hits – indeed, did not reach significance for Hits alone – whereas in our previous experiment, the effect was significant for both Hits and False Alarms (Taylor and Henson, in press). We have previously found a trend for a greater effect of repetition primes on K-False Alarms than K Hits (Woollams et al., 2008), but an informal review of published results using the Jacoby and Whitehouse paradigm would suggest that repetition primes affect studied as well as unstudied items, in which case, our present lack of effect on K Hits is likely to be a Type II error.

A second detail concerned a difference between the behavioral and fMRI results: Whereas there was a greater increase in the number of R than K judgments for conceptually primed relative to unprimed trials, there was no such interaction between Memory Judgment and Priming Type in the BOLD signal in the “recollection” fROIs. Rather, the pattern across these parietal fROIs in Fig. 5B reflected a significant conceptual priming effect for R judgments, but a conceptual priming effect that was numerically larger, but just not significant, for K judgments (though this conceptual-K effect appeared to be driven by an outlier; see Footnote 4). This lack of a significant interaction in the fMRI data is probably the weakest part of the present argument that conceptual primes selectively increase recollection, so would deserve replication, with greater power (e.g., greater number of K judgments). Indeed, more generally, the incidence of R judgments (63% of all trials) was slightly higher than we expected on the basis of previous experiments (cf. 58% in Taylor and Henson, in press; 52% in Woollams et al., 2008), likely reducing the incidence of K judgments, and possibly reflecting an atypical sample (or a facilitatory effect on attention/memory of being in an MRI scanner!). Importantly, however, the finding that the correlation between the sizes of behavioral and fMRI conceptual “priming” across participants was significant for R judgments, but not for K judgments, reinforces a role of the parietal regions in conceptual priming that is specific to recollection (given that the significance of this correlation is independent of the presence or absence of any mean priming effects on R and/or K judgments).

The findings of conceptual priming effects in parietal fROI responses to R judgments, and in particular, the correlation of these BOLD effects and behavioral priming effects across participants, support the hypothesis that such primes increase recollection, but they do not speak to the particular cognitive mechanism(s) that underlie this effect. The medial and lateral parietal regions that were activated by our comparison of R versus K judgments have also been previously associated with recollection using a variety of paradigms, including source retrieval, not just the Remember/Know paradigm (Wagner et al., 2005). One possibility we have proposed (Taylor and Henson, in press; also raised in the Introduction above) is that conceptual primes subliminally reactivate semantically related information that had been spontaneously generated at Study, thereby increasing the probability of retrieval of “internal” source (Johnson et al., 1993). Such reactivation of internal source information could explain why the effect of conceptual primes is restricted to studied items (Hits), contrary to fluency-attribution accounts that have been used to explain the increase in K responses (Hits *and* False Alarms) following repetition primes. Further support for this hypothesis awaits future study.

It should be noted that a recollection-based interpretation of the parietal fROI results is neither necessary nor sufficient. It is not necessary because there may be another interpretation, other than recollection *per se*, for the increase in parietal BOLD signal (e.g., attention to internally- vs externally-generated information; Cabeza et al., 2008). This could be tested by use of other memory judgments, such as objective measures of internal versus external source information. The recollection hypothesis is not sufficient either because other behavioral findings in our previous studies remain to be explained. For example, this hypothesis does not explain why we have been unable to replicate the effect of conceptual primes on R judgments when using only conceptual primes throughout the experiment (i.e., without concurrent blocks of repetition primes; Taylor and Henson, in press).^6^ Rather, this latter finding would seem easier to explain in terms of the “artifact” hypothesis raised in the Introduction: that participants need to experience two different types of fluency, in conjunction with being required to give mutually-exclusive R/K judgments, in order for R judgments to be affected. The latter could be tested simply by repeating the above experiments, complete with fMRI, but using independent ratings of remembering and knowing (Higham and Vokey, 2004; Brown and Bodner, 2011; Kurilla and Westerman, 2008).

Importantly, however, the recollection hypothesis is clearly productive, in terms of predictions for future experiments. One test, for example, would be to manipulate the study task: Only when that task is “deep” enough to engender semantic elaboration (as likely for the “interestingness” task used here), should the effect of conceptual primes on R judgments occur (i.e., no effect should be found when the Study task focuses on non-semantic features such as phonology/orthographics).^7^ More specifically, manipulation of the likelihood that the prime *in particular* is generated as a semantic associate at Study should produce modulation of the conceptual priming effect on R, but not of the repetition priming effect on K. Further convergence might come from considering paradigms in which semantic manipulations lead to false recollection, such as the Deese–Roediger–McDermott (DRM) paradigm (Deese, 1959; Roediger and McDermott, 1995), in which conceptual fluency arising from (studied) associates of the (unstudied) target can be misattributed to memory, resulting in false recollection of the target.

Finally, note that the two types of prime did differ in post-experimental testing of the prime visibility, with forced-choice performance being above chance for conceptual primes (and unrelated primes), but not repetition primes. This is expected, because the perceptual overlap between Repetition primes and targets is relatively large (the same word in different case), which results in the target more effectively masking the prime. In the present procedure, however, it is impossible to say whether this difference in prime visibility (when participants are explicitly directed toward the primes) accurately reflects prime visibility during Test blocks, and whether such visibility actually affected priming in the main experiment. *Intentional* identification of masked repetition primes during a recognition memory test has been shown to increase “old” responses, and in particular, false-alarm R responses (Higham and Vokey, 2000, 2004), but it is unknown whether this effect extends to *incidental* identification of primes, which is difficult to measure. In the present study, it is likely that the Visibility Test overestimates visibility during the memory test: Attention is focused on identifying the prime rather than on retrieving memories related to the target, and the forced-choice nature of the test allows participants to guess based on partial information or to focus on single letters or features, which may explain the improvement in performance when the prime differs from the target. Indeed, participants who report no awareness of primes after the experiment routinely perform above chance on the Visibility Test.

Therefore, an arguably better estimate of whether primes were visible during Memory Test blocks is simply the participants' self-reported awareness of “hidden words”. In our experiments, typically fewer than half of the participants report awareness of prime words during the experiment, and fewer still report that they were able to identify prime words on some trials (the rest say they saw “something” that may have been a word). Contrary to the notion that awareness of primes causes the (differential) priming effects, participants who report no awareness of the masked prime words (pooled from the present study and Taylor and Henson, in press, in order to increase power), the same pattern of results obtains: Conceptual priming increases R and Repetition priming increases K (analysis and results described in Taylor and Henson, in press). This suggests that awareness of “something” being flashed before a test item is not an important factor in the present priming effects (see also Klinger, 2001). Further, this null effect of awareness is consistent with Joordens and Merikle’s (1992) finding that brief masked primes (57 msec) produce the Jacoby–Whitehouse effect whether participants are told of the primes' existence in advance (“aware” instructions) or not (“unaware instructions”).

### 4.2 Integration with previous fMRI results

While previous fMRI studies have implicated the hippocampus as well as parietal cortex in recollection, we did not find activity in hippocampus for the R Hit > K Hit comparison that survived whole-brain correction (though it is likely to have had survived correction for a smaller search space, e.g., hippocampi alone). Nonetheless, the hippocampus was clearly identified by the CR > K Hit comparison, and further examination suggested that it also showed greater activity for R Hits than K Hits. Indeed, the U-shaped pattern across R Hit, K Hit and CR judgment types has been observed in numerous previous fMRI studies, and often interpreted in terms of hippocampal involvement in both (1) the recollection of studied items and (2) the encoding of novel, unstudied items (with evidence of the latter occurring even during a recognition memory test; Buckner et al., 2011; Stark and Okado, 2003). Indeed, using intracranial electroencephalography (EEG) during a recognition memory test, we have recently found both recollective and novelty effects in hippocampus, but with different latencies (Staresina et al., 2012): An early, pre-recognition-decision recollection effect and a later, post-recognition-decision novelty effect, which would simply summate to produce the U-shaped pattern in the magnitude of the BOLD response (at least, using the standard fMRI analysis employed here). The present fMRI findings reinforce these previous findings, and go further in that the lack of an effect of conceptual priming in hippocampus, in contrast with that found in the parietal regions, further supports a functional dissociation between the roles of hippocampus and parietal cortices during recollection/recall (Ramponi et al., 2011).

The regions showing greater BOLD responses for K Hits than Correct Rejections are broadly consistent with many previous fMRI studies of the basic “old-new” effect, particularly in that they appeared to be driven by the distinction between Hits and Correct Rejections, rather than between Remembering and Knowing. Most notable in this respect are the more superior parietal regions, which concur with many previous dissociations between inferior and superior parietal activations during recognition memory (Wagner et al., 2005; Cabeza et al., 2008). Nonetheless, it should be noted that Hits and Correct Rejections differ not only in the study status of the target item, but also in the “old-new” decision given (and possibly perceived “targetness”; Herron et al., 2004). The implications of this different “old-new” decision are particularly important in the present paradigm, given that only “old” decisions required a further R/K judgment. Though we attempted to match the visual and motor requirements of the R/K judgment with those following “new” decisions, by also requiring a second (left/right) judgment after a “new” decision, these second judgments were unlikely to be matched in terms of RT, overall “difficulty”, etc (and the estimated BOLD response is likely to include contributions from both decisions within each trial, due to their temporal proximity). This may explain some of the prefrontal differences between K Hits and CRs. Nonetheless, it is interesting to note that we did not see any regions that showed evidence of greater activity for K Hits than R Hits, unlike a previous study of ours (Henson et al., 1999), which found several prefrontal regions that were more active for K Hits than R Hits. That study used only a single, three-way R–K–New judgment however (i.e., a one-step rather than two-step R/K method, Eldridge et al., 2000; Knowlton and Squire, 1995), and one possibility is that the present two-step method offered better matching of the executive processes entailed by each decision (or rendered the R/K judgment less likely to be re-mapped to confidence; Henson et al., 2000).

Finally, it is surprising that we did not detect any effects of masked repetition priming, at least that survived whole-brain correction. We have found a reliable ERP effect of repetition priming within a very similar paradigm (Woollams et al., 2008), though it is possible that this effect is too small/transient to be easily detected with a hemodynamic measure like BOLD. Nonetheless, others have reported BOLD effects of masked repetition priming of visual words (though in a different task; Dehaene et al., 2001) in ventral temporal regions, and it is interesting to note that, at an uncorrected threshold of *p* < .001, we did see a cluster of nine voxels in left anterior ventral temporal cortex [with peak coordinates (−33 −30 −24)] that showed a repetition priming effect. Indeed, this region showed reduced BOLD responses for primed relative to unprimed trials in the Repetition condition, but not in the Conceptual priming condition, which is consistent with a lexical/phonological/orthographic (i.e., pre-semantic) fluency signal, and this response reduction appeared unmodulated by Memory Judgment, consistent with our ERP effect (Woollams et al., 2008). The potential role of this masked “repetition suppression” effect during recognition memory tests clearly deserves further investigation.

### 4.3 Caveats with present analyses

Finally, several caveats should be noted when relating our fMRI and behavioral analyses. Foremost, the behavioral priming effect is measured by the *number* of trials given an R or K judgment, whereas the fMRI priming effects reflect the *mean* BOLD signal per trial with an R or K judgment, which was furthermore restricted to *studied* trials. Thus not only is the fMRI R/K data restricted to Hits, but logically one could find a difference in the number of R/K judgments (behaviorally), in the absence of any difference in the mean BOLD signal whenever an R/K judgment is actually given (in this sense, the BOLD signal is more like the mean RT per judgment type). We restricted fMRI analysis to Hits because this has been conventional in this field (as well as in ERP research), and has the advantage of controlling for other confounding differences between Hits and, say, Misses, for example in terms of a different “old/new” key press. It would be possible to estimate the mean BOLD response to all primed and all unprimed trials, regardless of R/K judgment type or of study status, which might identify brain regions whose activity correlates with the number of R/K judgments given (and hence be more comparable to the present behavioral measure of priming). The downside of this type of analysis however, as noted above, would be that any such differences between primed and unprimed trials (or correlations across participants) could reflect trivial differences in the number of trials given a specific key press, rather than the number of trials associated with recollection versus familiarity *per se*, or with correct versus incorrect recognition memory.

A second caveat concerns how we identified brain regions associated with recollection/familiarity. The appropriate comparison of experimental conditions actually depends on the hypothetical relationship between recollection and familiarity: Whether they are redundant, independent or exclusive (Knowlton and Squire, 1995; Mayes et al., 2007). By contrasting R Hits with K Hits to isolate recollection, we have implicitly assumed that recollection is redundant with familiarity (i.e., that familiarity always co-occurs with recollection, so can be canceled by subtracting K Hits from R Hits). If however recollection and familiarity are mutually exclusive, then any activations found for R Hits versus K Hits could reflect either increased activity associated with recollection, or decreased activity associated with familiarity. In this case, an arguably more appropriate contrast would be R Hits versus Correct Rejections to isolate recollection (and K Hits versus Correct Rejections to isolate familiarity). Or if recollection and familiarity are independent, then an appropriate test for recollection might be the conjunction of a difference between R Hits versus Correct Rejections, but no difference between K Hits and Correct Rejections (while the contrast for familiarity would be the conjunction of a difference between K Hits versus Correct Rejections, but no difference between R Hits and Correct Rejections). We have not explored these other alternatives here, since our aim was to isolate recollection (less so familiarity), and the fact remains that the parietal regions we found for our comparison of R Hits versus K Hits concur with many previous neuroimaging studies that have used other procedures (such as objective measures of source memory). Nonetheless, these are examples of generic considerations that need to be kept in mind when interpreting fMRI data on recollection and familiarity.

## Figures and Tables

**Table 1:** Behavioral data: Mean (min–max range) percentage of trials for each type of Prime (Conceptual *vs* Repetition,/60) that were given each type of Memory Judgment (R, K, New) for each Prime Status (Primed, Unprimed) and Study Status (Studied, Unstudied).

Memory judgment	Conceptual				Repetition			
Studied		Unstudied		Studied		Unstudied	
Primed	Unprimed	Primed	Unprimed	Primed	Unprimed	Primed	Unprimed
R	62.1 (28.3–96.7)	57.3 (21.7–100)	3.6 (0.0–16.7)	3.7 (0.0–21.7)	61.6 (11.7–95.0)	60.8 (10.0–93.3)	3.3 (0.0–11.7)	2.6 (0.0–13.3)
K	16.9 (0.0–51.7)	18.9 (0.0–51.7)	12.6 (0.0–33.3)	15.5 (0.0–38.3)	16.7 (0.0–41.7)	17.2 (0.0–43.3)	21.8 (3.3–51.7)	12.2 (0.0–33.3)
New	18.4 (1.7–40.0)	22.0 (0.0–53.3)	82.6 (51.7–98.3)	79.4 (46.7–100)	19.3 (1.7–66.7)	20.2 (0.0–71.7)	74.2 (36.7–93.3)	84.5 (53.3–98.3)

**Table 2:** Clusters and their peaks for main contrasts of Memory Judgment, *p* < .05 FWE whole-brain corrected. Within each cluster, total cluster volume (voxels) and coordinates of the global peak are shown in ***bold***; coordinates of any local peaks more than 8 mm apart are listed beneath in regular font weight. Note: no voxels survived correction for contrast of K Hit > R Hit.

Region	Voxels	MNI Coordinates			*Z*-score
X	Y	Z
R Hit > K Hit					
Left inferior parietal	**245**	**−48**	**−57**	**+36**	**6.15**
	−36	−69	+36	5.85
Bilateral medial parietal Posterior cingulate	**244**	**+12**	**−45**	**+33**	**5.67**
	−9	−45	+30	5.02
	−6	−30	+42	5.02
	−9	−69	+33	5.48
Right inferior parietal	**18**	**+39**	**−66**	**+30**	**5.06**
K Hit > R Hit (nothing)					
K Hit > CR					
Left superior parietal	**33**	**−39**	**−51**	**+45**	**5.52**
Left medial parietal	**25**	**−9**	**−69**	**+36**	**6.17**
Bilateral anterior cingulate	**188**	**−6**	**+24**	**+42**	**6.99**
Left anterior insula	**67**	**−30**	**+21**	**−3**	**6.47**
Right anterior insula	**22**	**+33**	**+21**	**−9**	**5.52**
CR > K Hit					
Left anterior hippocampus	**8**	**−27**	**−9**	**−18**	**5.13**

## Footnotes

1 We actually used the judgment label “Familiar” rather than “Know”, because in a number of studies, we have found that some participants are confused by the greater confidence implied by colloquial usage of “know” compared with “remember”. Nonetheless, we maintain the “K” (and hence “R/K”) label in this paper for consistency with previous studies.

2 An analysis of priming for K *without* independence assumptions produced the same pattern of results: Masked Conceptual primes did not affect K judgments [*t*(21) = −1.75, *p* = .09]; whereas masked Repetition primes increased K judgments [*t*(21) = 2.61, *p* < .05].

3 For non-independence K: Masked Repetition primes increased K FAs [*t*(21) = 4.27, *p* < .001] but not K Hits (*t* < 1).

4 The numerically larger Conceptual priming effect for K than for R, and associated large standard error, was driven by a single outlier (value: 2.38; see Figs. 5B, 6B), and removal of this outlier reduced the K effect from .14 to .01 (whereas R changed from .10 to .09), producing a pattern consistent with the behavioral results (i.e., conceptual priming of R but not K). However, removal of this outlier based on the data of interest would be circular, and the participant was not an outlier on other (independent) measures.

5 Given that the Unprimed conditions of Repetition and Conceptual Prime Types do not differ logically, this main effect of Priming Type, in the absence of an interaction with Prime Status, is difficult to interpret. It could reflect a “state” effect – i.e., a difference in the way that both primed and unprimed trials are processed as a function of the nature of the priming block – or it may simply be a type I error. In either case, we do deem it worthy of further discussion here.

6 The recollection hypothesis also seems difficult to reconcile with previous demonstrations that associatively related primes, rather than the present semantically related primes, increase K rather than R judgments (Rajaram and Geraci, 2000). Such strong associates of target words would also seem likely to be generated at study, though this is likely to depend on the precise study task (explicit memorization in Rajaram and Geraci, 2000). Note however that the semantic primes used by Rajaram and Geraci (2000) were not masked, so were likely to be clearly visible to participants (unlike here), which may well have affected how the participants attributed any conceptual fluency, and hence also possibly explain this difference between our and their study.

7 Though the type of study task may also affect the extent to which source information is “unitized” with the target item: When the study task enables such unitization, the effect of conceptual primes may be experienced as familiarity rather than recollection (Kurilla, 2011).
